# Joint Multimodal Embedding and Backtracking Search in Vision-and-Language Navigation

**DOI:** 10.3390/s21031012

**Published:** 2021-02-02

**Authors:** Jisu Hwang, Incheol Kim

**Affiliations:** Department of Computer Science, Kyonggi University, Suwon-si 16227, Korea; rabbit0304@kyonggi.ac.kr

**Keywords:** multimodal embedding, natural language instruction, panoramic image, vision-and-language navigation task, deep neural network, pretrained model, three-dimensional simulated indoor environment, backtracking-enabled greedy local search

## Abstract

Due to the development of computer vision and natural language processing technologies in recent years, there has been a growing interest in multimodal intelligent tasks that require the ability to concurrently understand various forms of input data such as images and text. Vision-and-language navigation (VLN) require the alignment and grounding of multimodal input data to enable real-time perception of the task status on panoramic images and natural language instruction. This study proposes a novel deep neural network model (JMEBS), with joint multimodal embedding and backtracking search for VLN tasks. The proposed JMEBS model uses a transformer-based joint multimodal embedding module. JMEBS uses both multimodal context and temporal context. It also employs backtracking-enabled greedy local search (BGLS), a novel algorithm with a backtracking feature designed to improve the task success rate and optimize the navigation path, based on the local and global scores related to candidate actions. A novel global scoring method is also used for performance improvement by comparing the partial trajectories searched thus far with a plurality of natural language instructions. The performance of the proposed model on various operations was then experimentally demonstrated and compared with other models using the Matterport3D Simulator and room-to-room (R2R) benchmark datasets.

## 1. Introduction

Driven by the rapid growth of computer vision and natural language processing technologies, in recent years there has been a growing interest in multimodal intelligent tasks that require the ability to concurrently understand various forms of input data such as images and text. Some of the typical multimodal intelligent tasks are vision question answering (VQA) [1] that generate answers to natural language questions on the image presented, visual dialog [2] that holds a meaningful question and answer (Q&A) dialog on the input image, and image/video captioning that generates texts describing the contents of the input image or video. More advanced multimodal intelligent tasks have also been presented, including embodied question answering (EQA) [3], assuming an embodied agent moving around in a virtual environment [3], interactive question answering (IQA) [4], cooperative vision and dialog navigation (CVDN) [5], remote embodied visual referring expression in real indoor environments (REVERIE) [6], and vision and language navigation (VLN) [7].

**Figure 1:**
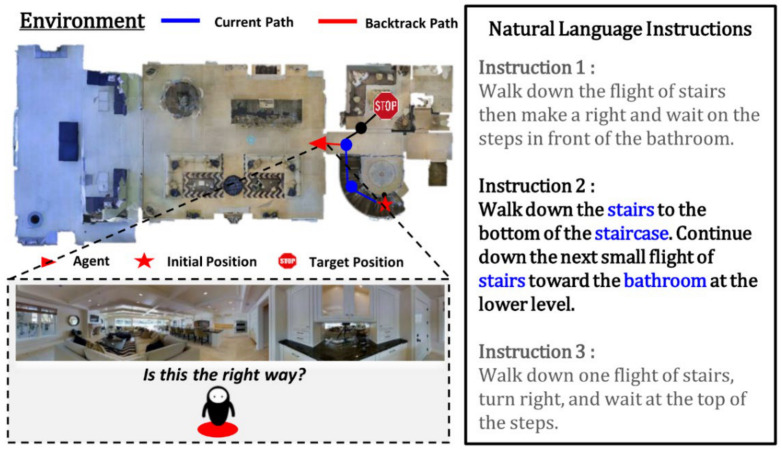
Examples of vision and language navigation (VLN) tasks.

Among these multimodal intelligent tasks, we will explore VLN tasks, in which an agent moving in a three-dimensional virtual environment is requested to navigate to a particular location following natural language instructions. Consider an agent placed in a 3D virtual indoor environment generated by the Matterport3D Simulator [8]. As shown in the example presented in Figure 1, a VLN task requires the agent to progress to the target position, relying on the natural language instructions and visual cues of the environment corresponding to the agent’s vision. For model training and testing, we considered room-to-room (R2R) [7] datasets. Datasets for each navigation task include an optimal path from a specific initial position to the target position within the environment and a set of three different natural language instructions that describe the optimal path.

As shown in Figure 1, the natural language instructions for a VLN task contain instructions that request the agent to perform high-level actions such as “walk down” and “continue down” around various landmarks (e.g., bathroom and stairs) within the environment. Therefore, the VLN agent needs to solve many problems: it needs to acquire knowledge of its location in the current environment to determine the next action; it needs to identify the landmark in the input image mentioned in the instruction; and it needs to choose the low-level action to implement the high-level action in accordance with the instruction. In particular, the agent should be able to perform alignment and grounding of multimodal input data to understand the natural language instructions coherently in relation to the real-time input images.

Because most natural language instructions provide only partial descriptions of the trajectory to the target position, the agent encounters difficulties in understanding the instructions unless they are efficiently combined with visual features using the alignment and grounding feature. In VLN-related studies [7,9,10,11,12,13,14,15,16,17,18,19,20,21], various attention mechanisms, such as visual or textual attention, are employed to ensure the alignment and grounding between natural language instruction and input images. However, these attention-based VLN models are trained only to learn the association between natural language instructions and images with a limited number of R2R datasets; moreover, they have difficulties acquiring broader general knowledge of the relationship between natural language instruction and images. Therefore, it is a great challenge for attention-based VLN models to extract sufficient context from multimodal input data, including natural language instructions and images, to make real-time action decisions. To address this drawback, researchers proposed transformer-based pretrained models [20,21]. Unlike attention-based models, pretrained models are equipped with feature aligning natural language instructions and images, trained with attended masked language modeling (AMKM) and action prediction (AP) tasks based on a transformer neural network before being used for VLN tasks. However, current pretrained models have insufficient capacity to reflect the wealth of statuses and actions experienced by the agent in the past.

Effective path planning and action selection strategies for reaching the target position are crucial for executing VLN tasks. Most VLN-related studies [7,9,10,11,12,13,14,15,16,17,18,19,20,21] applied search techniques that rely on local scoring of candidate actions, such as greedy local and beam searches. In the case of the greedy area search, the search speed is increased because the search range is limited; however, its success rate is low because it is difficult to return to the original path once a wrong path is selected. Beam search, developed in an attempt to overcome this problem, has a higher task success rate compared with the greedy area search owing to the concurrent search of several candidate paths in parallel. However, its search efficiency is low considering the total length of the search path to be explored in parallel. It is also associated with implementation problems because one agent cannot simultaneously search multiple paths in parallel in an actual navigation environment.

In an effort to overcome the limitations of previous studies for solving VLN tasks, we propose the joint multimodal embedding and backtracking search (JMEBS), a novel deep neural network model. The proposed model uses a transformer-based, joint multimodal embedding module to obtain a text context that is efficient for action selection based on natural language instructions and real-time input images. Unlike the conventional transformer-based pretrained models, the JMEBS model uses multimodal context at each time step that connects the current image to natural language instructions, and temporal context that contains the agent’s past statuses and actions. One of its salient features is that the context information extracted by the module can be integrated into various path planning and action selection strategies. It is also equipped with a backtracking-enabled local search feature designed to improve the task success rate and optimize the navigation path based on the local and global scores related to candidate actions. Additionally, a global scoring method is used to compare partial trajectories searched with various natural language instructions. In summary, our contributions include:

- A novel deep neural network model, with joint multimodal embedding and backtracking search for VLN tasks.
- Rich context utilization based on a transformer-based multimodal embedding module and a temporal contextualization module.
- A backtracking-enabled greedy local search algorithm to improve the success rate and optimize the path.
- A novel global scoring method to monitor task progress by comparing the partial trajectories searched thus far with a plurality of natural language instructions.
- State-of-the-art results on the VLN task.

The remainder of this article is organized as follows: Section 2 presents an overview of the related literature. Section 3 provides a detailed explanation of the design of the proposed JMEBS model. Section 4 presents the JMEBS implementation environment and the results of various experiments using R2R benchmark datasets. Section 5 draws conclusions and presents directions for future research.

## 2. Related Works

In a study on VLN tasks [7], a relatively simple deep neural network model of the sequence-to-sequence (Seq2Seq) type was proposed, in which an action sequence was output from two input sequences with input video stream and natural language instructions, respectively. A few other VLN-related studies [9,15,16] presented methods to solve the problem of insufficient R2R datasets for training VLN models. They undertook various data augmentation techniques, including the development of a speaker module to generate additional training data [9], new training data through environment dropout (eliminating selected objects from the environment) [15], and more sophisticated task data by concatenating the existing R2R data [16].

Researchers have attempted to understand the association between natural language instructions and real-time input images in a range of studies on VLN tasks. One of these attempts resulted in an attention mechanism for co-grounding multimodal input data [9,10,11]. In particular, Object-and-Action Aware Model (OAAM) [10] separated object-centered instruction from action-centered one, and then matches them to their own counterpart visual perception and action orientation. Another work [11] proposed a relationship graph for modeling the inter-modal relationships between natural language instruction and input image, and the intra-modal relationships among visual entities. These models used an end-to-end training to train the association between the words appearing in natural language instructions and input images. However, only R2R datasets with limited range were used. This training mechanism is thus unsuitable for training comprehensive vision–language knowledge. Another drawback of the models is the use of a recurrent neural network to model the sequence of words used in natural language instructions, which is unsuitable for parallel processing. To overcome these limitations, some researchers developed pretrained models [20,21] in which natural language instructions and images for the VLN task are embedded together with large-scale benchmark datasets in addition to R2R datasets. VisualBERT [22], Vision-and-Language BERT (ViLBERT) [23], Visual-Linguistic BERT (VL-BERT) [24], and UNiversal Image-TExt Representation (UNITER) [25], are pretrained models applicable to various vision–language tasks. There are also models pretrained specifically for VLN tasks [20,21]. These VLN-specific models have a simple structure that immediately selects one of the candidate actions because they use only the multimodal context of the concurrently embedded data extracted according to natural language instructions and input images. Therefore, these pretrained VLN models cannot sufficiently use the temporal context reflecting the statuses and actions experienced by the agent in the past, nor can they deviate from the built-in greedy local search to combine with other search approaches. To overcome this limitation, we developed the JMEBS model using the pretrained model proposed in [20] as a joint embedding module to obtain a multimodal context. By adding the temporal contextualization module, the JMEBS model is designed to enable the agent to select actions by taking into account its own past statuses and actions. Moreover, by adopting the pretrained model of [20] as an independent module, the JMEBS model can be easily combined with a novel greedy local search capable of conducting backtracking actions.

Conversely, one of the most important issues associated with the solution of a VLN task is an efficient path planning and action selection strategy. In a VLN task, the agent is not explicitly given the target position; it is given only the natural language instructions that provide directions to the target position. Hence, the agent needs to perform efficient path planning and action selection strategies to reach the target position based on the real-time input image and natural language instructions. In a VLN task, a graphic map is predefined for each environment, and the agent should move from one node to the next to navigate to the target position. Therefore, in previous studies, greedy local [7,9,10] and beam searches [14], which were suitable for graph searches, were mostly used for VLN tasks. In general, a greedy local search—a technique for searching the node with the highest local score among adjacent nodes of the current node at each time step—has high-search efficiency owing to its narrow search range. However, it has a high-task failure rate once a false path is selected during the search. Beam search, developed in an attempt to overcome the drawback of greedy local search, has a higher task success rate compared to the greedy area search owing to the concurrent search of k-number of candidate paths in parallel. However, it has low practicability given that one agent cannot simultaneously search multiple paths in parallel in an actual navigation environment, and low search efficiency, considering the total length of the search path to be explored in parallel.

Attempts to overcome these drawbacks of greedy local search and beam search have been made in two VLN-related studies [13,14]. In [13], an extended local search, including rollback action, was proposed, using a progress monitor for the position of each action with respect to the target position as heuristics for search. In [14], a new frontier-aware search with a backtracking (FAST) search algorithm or FAST navigator was proposed that upgraded the efficiency of the beam search. The FAST navigator can backtrack if it detects a mistake by simultaneously comparing partial paths of different lengths using local and global information. Going a step further, the proposed model JMEBS employs a backtracking-enabled greedy local search (BGLS), which is similar to rollback [13] or FAST [14] in that the agent is backtracking-enabled, but uses a different local and global scoring strategy for action selection. The JMEBS model performs local scoring using the multimodal context extracted from the natural language instruction, real-time image, and the temporal context reflecting the agents’ experience over time. Furthermore, its global scoring involves a comparison of all partial trajectories of each point visited from the initial position to the current position using a new scoring function.

## 3. Materials and Methods

### 3.1. Problem Description

**Figure 2:**
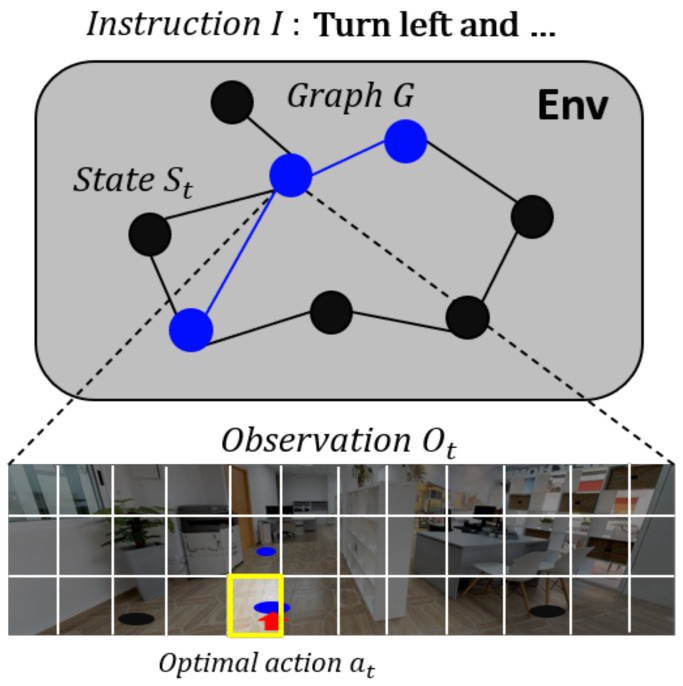
Illustration of the VLN environment.

The VLN is a task in which the agent navigates to an unknown target position following natural language instructions. It is assumed that the real-time input of the panoramic image of the environment is provided to the agent that performs the task. Figure 2 illustrates the VLN task environment.

As shown in Figure 2 (top), a graphic topological map (*G*) consisting of nodes (waypoints) and edges (connections) is defined at each position visited by the agent. The VLN server controls the agent to navigate only on *G*. In this way, it is not exposed to the agent that determines each navigation action by perceiving the environment. As shown in Figure 2 (bottom), the agent must estimate its own position and the composition of the environment by relying on the 360° panoramic image (observation *O_t_*) acquired at the current position (state *S_t_*) at each time step. Accordingly, the panoramic image is divided into 36 partial images of equal size, each of which is a candidate action. If the agent selects and executes any of these candidate actions, the management system moves the agent to the node closest to the chosen direction on *G*. The agent navigates by repeating this process and performs the “stop” action at the position predicted as the goal. If the agent’s final position is within 3 m of the target position, the management system marks the task as successful.

### 3.2. Proposed Model

The proposed model JMEBS performs the local scoring of a candidate action based on the multimodal context extracted from the natural language instruction and panoramic image using the pretrained joint embedding module and temporal contextualization reflecting past statuses and actions. Additionally, it performs global scoring to determine the need for backtracking by comparing the natural language instructions describing the entire trajectory from the initial to the final position with the partial trajectories up to the current position.

**Figure 3:**
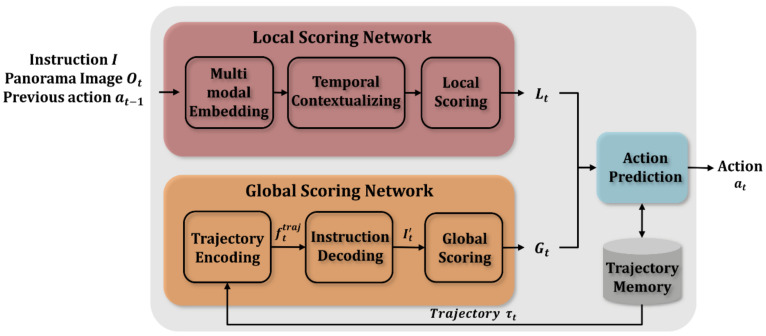
Overall architecture of the proposed model, JMEBS.

Accordingly, the JMEBS model is largely divided into a local scoring network, a global scoring network, and an action selection network, as shown in Figure 3. The global scoring network gives a local score to each of the candidate actions based on multimodal and temporal contexts. The agents select one of the candidate actions based on the local scores given to them and navigate. Finally, the global scoring network is used to determine the need for backtracking by comparing the natural language instructions describing the entire trajectory with the partial trajectories up to the current position.

The local scoring network consists of three stages: multimodal embedding, temporal contextualization, and local scoring. In the multimodal embedding stage, panoramic images and natural language instructions are embedded to enable the use of real-time input data. The joint multimodal embedding module proposed in this study is used in the multimodal embedding process to enable information exchange between the image and natural language modes. This process involves visual information embedding based on natural language instruction, and natural language embedding based on visual information. The temporal contextualization stage is composed of a context decoding module for updating temporal context and a co-attention module for detecting the focus area from the input data using the context feature. In the context updating stage, a new context $h_{t}$ is set using the input data (visual feature and previous action) delivered to the agent at each time step t from the environment and the previous context $h_{t-1}$. The co-attention stage includes the attention network for each mode that determines the area to focus on according to the instruction and image received based on context. Finally, in the local scoring stage, the actual candidate actions are rated. A detailed explanation of the local scoring stage is provided in Section 3.3.

The optimal action to take at the current time step (t) decided in the local scoring stage is stored in the trajectory memory wherein the entire trajectory sequence from the initial to the target position is stored. Furthermore, the global scoring network is used to determine whether to execute the selected action or rollback. The global scoring network is subdivided into three stages: the trajectory encoding stage to encode the stored trajectory; the instruction decoding stage to generate natural language instructions to describe the trajectory; and the global scoring stage that requires a process step to check the trajectories generated thus far against the ground truth trajectories. Given that the agent is not provided the ground truth trajectories owing to the nature of the VLN tasks, scoring is performed by generating the instructions describing the trajectories executed up to the current position and checking them against the ground truth instructions. A detailed explanation of the local scoring stage is provided in Section 3.4.

### 3.3. Local Scoring

**Figure 4:**
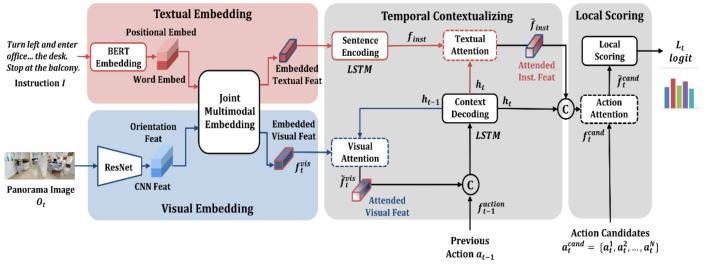
Architecture of local scoring network.

Figure 4 illustrates the local scoring network that rates candidate actions based on multimodal and temporal contexts. At each time step t, the agent takes action according to the instruction I that describes the entire trajectory, panoramic image $O_{t}$, previous action$\;a_{t-1}$, and candidate actions $a_{t}^{cand}$ to choose from at the current position. The local scoring network is subdivided into the multimodal embedding stage, temporal contextualization stage, and local scoring stage. The local scoring process comprises distinct processes for the generation of instruction features (red line) and visual features (blue line).

First, in the multimodal embedding stage, instruction I performs word embedding using pretrained BERT and positional embedding, including the positional information of the word. For the panoramic image, the convolutional neural network (CNN) visual features are based on the pretrained ResNet-152 and the agent’s orientation feature. The institutional embedding dataset for each mode is input as the joint multimodal embedding module so that embedding features can be generated based on intermodal data exchange.

**Figure 5:**
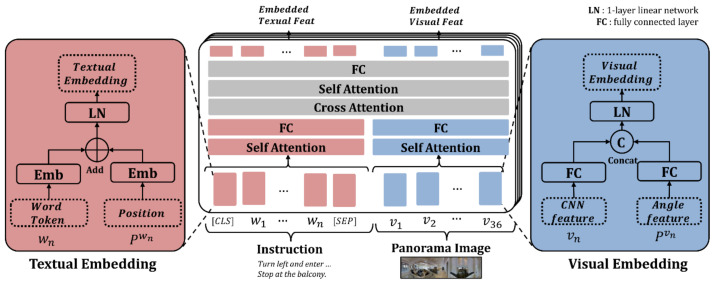
Architecture of joint multimodal embedding module.

Figure 5 illustrates the architecture of the joint multimodal embedding of the JMEBS. The joint embedding module is composed of a mode-specific embedding layer and transformer layers for intermodal exchange. The embedding layer accommodates the text embedder to embed the instructions and the visual embedder for processing the panoramic image. Textual embedding is performed to generate the embedding feature of each word by concatenating the BERT embedding data ($w_{n}$) of each word of instruction I and the positional embedding ($P^{w_{n}}$) of each word.

The visual embedding process uses two types of features: the image feature ($v_{n}$) of each of the 36 partial images extracted from the panoramic image with a CNN and the orientation feature ($P^{v_{n}}$, orientation feat) viewed from the agent’s current position. The orientation feature is the value corresponding to each of the 36 partial images, that is, a 128-dimensional directional vector based on real numbers in the form of $\left[sin\varphi ;cos\varphi ;\;sin\theta ;\cos\theta \right]$, where φ is the orientation of the horizontal field-of-view (heading), and θ is the vertical field-of-view (elevation). In this case, “;” denotes the features that are used in the concatenated state. Conclusively, the orientation feature and CNN image features are fused into the visual embedding feature depending on the agent’s current orientation. Finally, the embedded text string and visual feature undergo intermodal data exchange through the co-transformer module (gray area), resulting in the acquisition of the embedded textual feature reflection image data and the visual feature reflecting the natural language instruction data.

The proposed model JMEBS contains a temporal contextualization module, as shown in Figure 4. As mentioned previously, the temporal contextualization stage context decoding module for context updating and the co-attention module for detecting the focus area from the input data use the context feature. The co-attention module contains a mode-specific attention (visual or textual attention) network for searching visual and textual features. The context decoding module is a network based on the long short-term memory (LSTM) used to reflect the sequence feature at each time step; it enables the generation of a new context ($h_{t}$) that reflects the current input data by inputting the visual feature, previous action, and previous temporal context ($h_{t-1}$), as expressed by Equation (1):

(1) $$h_{t}=LSTM\left(\left[{\tilde{f}}_{t}^{vis};\;f_{t-1}^{action}\right],\;h_{t-1}\right)$$

Herein, the input visual feature should be updated using the time-step data best suited for the current status instead of the embedded visual feature. Therefore, visual attention is used as an element of the co-attention module, thereby applying the soft-attention mechanism expressed by Equation (2) to the existing visual feature ($f_{t}^{vis}$) with the help of the previous temporal context ($\;h_{t-1}$). This leads to the generation of attended visual features (${\tilde{f}}_{t}^{vis}$) reflecting the actual temporal context, where g denotes a multilayer neural network and $\alpha_{t}^{vis}$ denotes the textual attention weight for the visual feature:

(2) $$z_{t}^{\mathrm{vis}}={\left(W_{v}h_{t-1}\right)}^{⊤}g\left(f_{t}^{\mathrm{vis}}\right),{\;\alpha }_{t}^{\mathrm{vis}}=\mathrm{softmax}\left(z_{t}^{\mathrm{vis}}\right),\;{\tilde{f}}_{t}^{\mathrm{vis}}=\alpha_{t}^{\mathrm{vis}}f_{t}^{\mathrm{vis}}\;$$

The next process is sentence encoding in which the embedding feature of each word extracted based on the joint embedding process is subjected to the LSTM model to generate an instruction feature ($f_{inst}$) that reflects the word sequence feature. The instruction feature generated is used for candidate action scoring. It is designed to perform textual attention according to the current status to select the instructed action that corresponds to a specific focus area. The textural attention process is expressed by Equation (3), wherein the focus word is selected based on the images up to the current time step and the current context ($h_{t}$), where $\alpha_{t}^{inst}$ denotes the textual attention weight:

(3) $$z_{t}^{\mathrm{inst}}={\left(W_{x}h_{t}\right)}^{⊤},{\;\alpha }_{t}^{\mathrm{inst}}=\mathrm{softmax}\left(z_{t}^{\mathrm{inst}}\right)\;{\tilde{f}}_{\mathrm{inst}}=\alpha_{t}^{\mathrm{inst}}f_{\mathrm{inst}}$$

Subsequently, a new situation (${\tilde{h}}_{t}$) is generated, with the entire temporal context ($h_{t}$) and the attended instruction feature (${\tilde{f}}_{\mathrm{inst}}$) reflected in it, as expressed by Equation (4). This is the final process for generating the core feature, with all word data most closely associated with the current status and action decisions reflected in it. To compute the predefined candidate action at each position of the agent ($f_{t}^{cand}$) and the weight of each action using the situation data (${\tilde{h}}_{t})$, the same soft-attention process is executed as in Equation (3):

(4) $${\tilde{h}}_{t}=tanh\left(W\left[{\tilde{f}}_{inst};h_{t}\right]\right)$$

Using the attended candidate action feature ${\tilde{f}}_{t}^{cand}$, the local score logit for $a_{t}^{k}$ is computed based on Equation (5). In this candidate action scoring from the local perspective based on the agent’s current input data and situation, the action with the highest score is selected as the next action:

(5) $$L_{t}\left(a_{t}^{k}\right)=softmax\left(W_{a}{\tilde{h}}_{t}\;{\tilde{f}}_{t}^{cand}{}^{⊤}\right)$$

The function $Loss_{LSN}$ for the loss of the local scoring network is defined as the cross-entropy loss of action decision, as expressed by Equation (6), where $y_{t}^{GT}$ denotes the ground truth action and $p_{t}^{k}$ denotes the action probability for each candidate action $a_{t}^{k}$.

(6) $$Loss_{LSN}=\overset{T}{\underset{t\;=\;1}{\sum }}y_{t}^{GT}log\left(p_{t}^{k}\right)$$

### 3.4. Global Scoring

**Figure 6:**
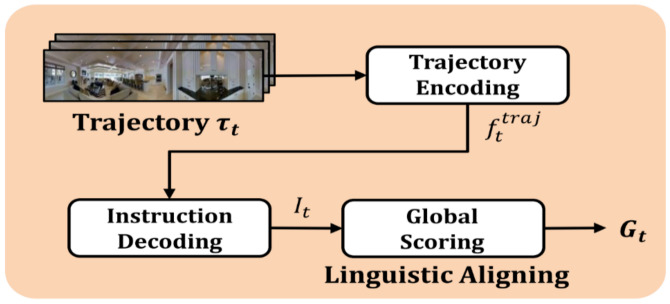
Architecture of global scoring network.

The global scoring network illustrated in Figure 6 determines the need for backtracking from a global perspective. The global scoring network operates in sync with trajectory memory (M), which stores the trajectories executed by the agent to reach the current position, as expressed by Equation (7):

(7) $$\tau_{t}=\langle s_{0},a_{0},\;s_{t}\rangle ,\;s_{k}\overset{a_{k}}{\to }s_{k+1}\;for\;0\leq k\leq t$$

An R2R dataset contains three different natural language instructions that describe the same trajectory of each VLN task. In the global scoring network, the extent to which the current trajectory is advancing toward the target position is assessed by checking the trajectories $\tau_{t}$ executed thus far against natural language instructions $I_{k}\in I,\;k=1,2,3$. To enable the comparison of the trajectories $\tau_{t}$ having a different mode (visual mode) with the natural language instructions I (textual mode), the speaker model [9] is used to transform $\tau_{t}$ to $I_{t}^{\prime }$. The final global scores are obtained by computing the similarity of the transformed instructions $I_{t}^{\prime }$ to the original instructions $I_{k}\in I,\;k=1,2,3$. To calculate the similarities among natural language instructions, we used dynamic time warping (DTW) in which a natural language instruction is considered a sequence of words, and the similarity between natural language instructions is calculated by applying the method of calculation of the similarity between two time-series data.

The global scoring network of JMEBS consists of three stages: trajectory encoding of the current trajectory $\tau_{t}$, instruction decoding to transform the current trajectory to natural language instruction $I_{t}^{\prime }$, and the final global scoring calculation. First, the trajectory $\tau_{t}$ is encoded as an image sequence-type trajectory vector $f_{t}^{traj}$ with the encoder LSTM, and then retransformed with an LSTM-based decoder to natural language instruction $I_{t}^{\prime }$ to match the trajectories executed to reach the current position. In the process of instruction decoding, we used the speaker model [9] with bidirectional LSTM (bi-LSTM) pretrained based on the instruction data in the existing R2R datasets.

In the final global scoring stage, the global score $G_{t}\;$of the action $a_{t}$ is calculated by comparing the trajectory transformed to the natural language $I_{t}^{\prime }$ with the three natural language instructions $I_{k}\in I,\;k=1,2,3$ provided by the R2R datasets, as expressed by Equation (8):

(8) $$G_{t}\left(a_{t-1}\right)=\frac{1}{3}\overset{3}{\underset{k\;=\;1}{\sum }}DTW\left(I_{k},\;I_{t}^{\prime }\right)$$

The loss function $Loss_{GSN}$ of the global scoring network is defined as the function of cross-entropy loss incurred in the course of trajectory encoding and instruction decoding, as expressed by Equation (9), where $z_{t}^{GT}$ denotes the ground truth word, and $w_{k}$ denotes the word probability for each candidate word constituting the instruction I.

(9) $$Loss_{GSN}=\overset{T}{\underset{t\;=\;1}{\sum }}z_{t}^{GT}log\left(w_{k}\right)$$

### 3.5. Backtracking-Enabled Greedy Local Search

The action selection strategy described in Algorithm 1 represents the BGLS algorithm used in JMEBS. In Algorithm 1, the algorithm is expressed as a function that receives input data ($O_{t}$ = image, I = natural language instruction, $a_{t-1}$ = previous action) and transforms them into output data (${\hat{a}}_{t}=$ the optimal action at the current position).
**Algorithm 1.** Pseudocode of backtracking-enabled greedy local search (BGLS) algorithm.**BGLS(**$O_{t},I,\;a_{t-1}$)1:  $\tau_{t}=\langle s_{0},\;s_{1},\ldots ,s_{t}\rangle \;\leftarrow Get\_Tarjctory\left(M\right)$
2: **if** ($Goal\left(s_{t}\right)$) **then return**
$a_{stop}$
3:  $G_{t}\left(a_{t-1}\right)\leftarrow Global\_Scoring$($a_{t-1},\;\tau_{t}$)4: **if** ($G_{t}\left(a_{t-1}\right)\leq G_{t-1}\left(a_{t-2}\right)$) **then**5:   $a_{back}\leftarrow Backtracking\left(s_{t}\right)$
6:    ${\hat{\tau }}_{t+1}\leftarrow Expand\_Trajectory(\tau_{t,\;}{\hat{s}}_{t+1}$)7:   $M\leftarrow Update\_Memory\left(M,\tau_{t+1}\right)$
8:   **return**
$a_{back}$
9:  **end if**10: **for each** possible action $a_{t}^{k}$ at state $s_{t}$
**do**11:   $L_{t}\left(a_{t}^{k}\right)\leftarrow Local\_Scoring\left(a_{t}^{k}\right)$
12: **end for**13: ${\hat{a}}_{t}\leftarrow argmax_{k\in K}\left(L_{t}\left(a_{t}^{k}\right)\right)$
14: ${\hat{s}}_{t+1}\leftarrow Update\_State\left(s_{t},{\hat{a}}_{t}\right)$
15: ${\hat{\tau }}_{t+1}\leftarrow Expand\_Trajectory(\tau_{t,\;}{\hat{s}}_{t+1}$)16: $M\leftarrow Update\_Memory\left(M,\tau_{t+1}\right)$
17: **return**
${\hat{a}}_{t}$


First, the trajectories executed thus far $\tau_{t}=\langle s_{0},a_{0},\;\ldots ,\;s_{t}\rangle$ are retrieved from trajectory memory M. If the current status $s_{t}$ is in the target position range $Goal\left(S_{t}\right)$, the stop action $a_{stop}$ is returned (lines 1–2 in Algorithm 1). Otherwise, the global scoring $Global\_Scoring\left(a_{t-1},\;\tau_{t}\right)\;$for the previous action $a_{t-1}$ is performed based on all the trajectories executed up to that time instant, $\tau_{t}=\langle s_{0},a_{0},\;\ldots ,\;s_{t}\rangle$ (line 3 in Algorithm 1). If the global score of the previous action $a_{t-1}$ has not improved compared with its previous action $a_{t-2}$, that is, $G_{t}\left(a_{t-1}\right)\leq G_{t-1}\left(a_{t-2}\right)$, the backtracking decision $Backtracking\left(s_{t}\right)$ is made to execute a rollback action $a_{back}\;$to the previous status $s_{t-1}$, and $Expand\_Trajectory\left(\tau_{t},\;s_{t+1}\right)$, that is, the expansion of the new trajectory $\tau_{t+1}$ to the status $s_{t+1}$ and $Update\_Memory\left(M,\;\tau_{t+1}\right)$ are decided, and the rollback action $a_{back}$ is returned (lines 4–8 in Algorithm 1). If the global score of the previous action $a_{t-1}$ has improved compared with its previous action $a_{t-2}$, that is, $G_{t}\left(a_{t-1}\right)>G_{t-1}\left(a_{t-2}\right)$, the local score of each candidate action $a_{t}^{k}$ available at the current status $s_{t}$ is calculated ($Local\_Scoring\left(a_{t}^{k}\right)$), and the action that has the highest local score ${\hat{a}}_{t}$ is selected (lines 9–10 in Algorithm 1). The new status ${\hat{s}}_{t+1}$, expanded as a result of the execution of the selected action ${\hat{a}}_{t}$, is updated to the new trajectory ${\hat{\tau }}_{t+1}\;Expand_{Trajectory\left(\tau_{t},\;{\hat{s}}_{t+1}\right)};$ the trajectory memory is updated $Update_{Memory\left(M,\;\tau_{t+1}\right)};$ and the action is returned (lines 11–14 in Algorithm 1).

As explained earlier, the new BGLS search algorithm available for both local and global scoring has a higher search efficiency compared with the conventional beam search algorithms and a higher task success rate compared with conventional greedy local search algorithms that have no possibility of backtracking.

## 4. Experiments

### 4.1. Dataset and Model Training

We conducted five different experiments to analyze the performance of the proposed model JMEBS using R2R datasets and the Matterport3D simulation environment. R2R [7] is a navigation dataset established by providing a trajectory for people to navigate the Matterport3D indoor environment from the starting point of a specific destination, and by collecting the natural language instructions that describe the trajectory. An R2R dataset contains three different natural language instructions describing each trajectory, and is configured to have training and validation data at a ratio of 2 to 1. JMEBS is implemented with the Python deep learning library Pytorch in the Ubuntu 16.04 LTS environment mounted with a Geforce GTX 1080Ti graphics processing unit. The performance assessment metrics used for the experiments are the navigation error (NE, m), success rate (SR, %), success rate weighted by path length (SPL, %), and trajectory length (TL, m). NE results are expressed as the mean distance between the agent’s final arrival point and the target location. SR represents the percentage of agent’s final positions less than 3m away from the target goal position. SPL considers the path length and success rate. The mean distance can be calculated using Equation (10):

(10) $$\frac{1}{N}\overset{N}{\underset{i\;=\;1}{\sum }}S_{i}\frac{l_{i}}{max\left(p_{i},\;l_{i}\right)}$$

For the model training, the learning rate was set to 0.1, the optimizer was the stochastic gradient descent (SGD), and the epoch was set to 50,000.

### 4.2. Experiments

**Figure 7:**
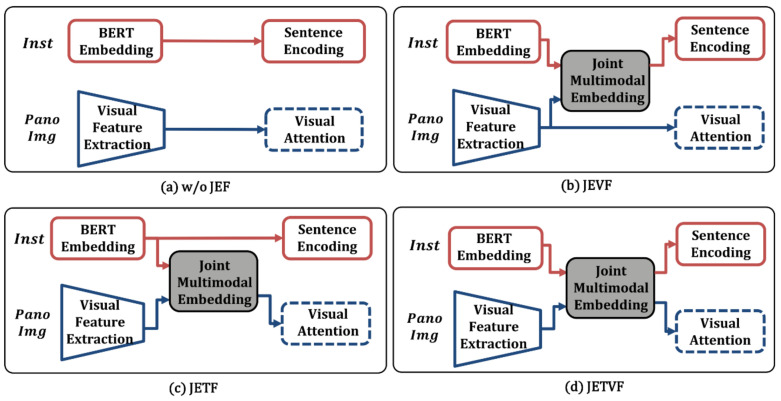
Different embedding features: (**a**) without joint embedding features (w/o JEF), (**b**) joint embedding visual features (JEVF), (**c**) joint embedding textual features (JETF), and (**d**) joint embedding textual and visual features (JETVF).

**Figure 8:**
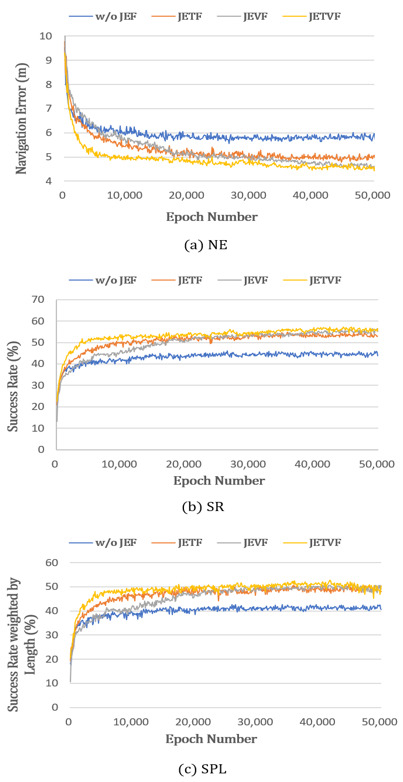
Comparison of performance using different embedding features: (**a**) navigation error (NE), (**b**) success rate (SR), and (**c**) success rate weighted by path lengths (SPL).

The first experiment aimed to prove the positive effect of the joint embedding textual and visual features (JETVF) employed in the proposed model. For this experiment, the JETVF are compared in three different conditions: (i) without joint embedding features (w/o JEF), (ii) only with joint embedding visual features (JEVF), and (iii) and only with joint embedding textual features (JETF). Figure 7a–d show the module w/o JEF, JEVF, JETF, and JETVF, respectively. The experimental results are presented in Table 1 and Figure 8.

The results presented in Table 1 show that the textual and visual features (JETVF) of JMEBS outperformed all other embedding features. Similarly, the case without joint embedding features (w/o JEF) was outperformed by all other cases. The case that used JETF generally scored slightly higher than the case that only used the visual features (JEVF). These findings verified the positive effect of joint multimodal embedding features on VLN tasks.

Figure 8 illustrates the epoch-dependent performance changes in relation to the use of different joint-embedding features. Figure 8a–c represent the performance levels measured by the NE, SR, and SPL, respectively. In the results of this experiment, the JETVF used in the JMEBS model had the lowest NE and highest SR and SPL values. Additionally, JETVF, that is, the case using both text and visual features, showed faster performance convergence than the other cases.

**Figure 9:**
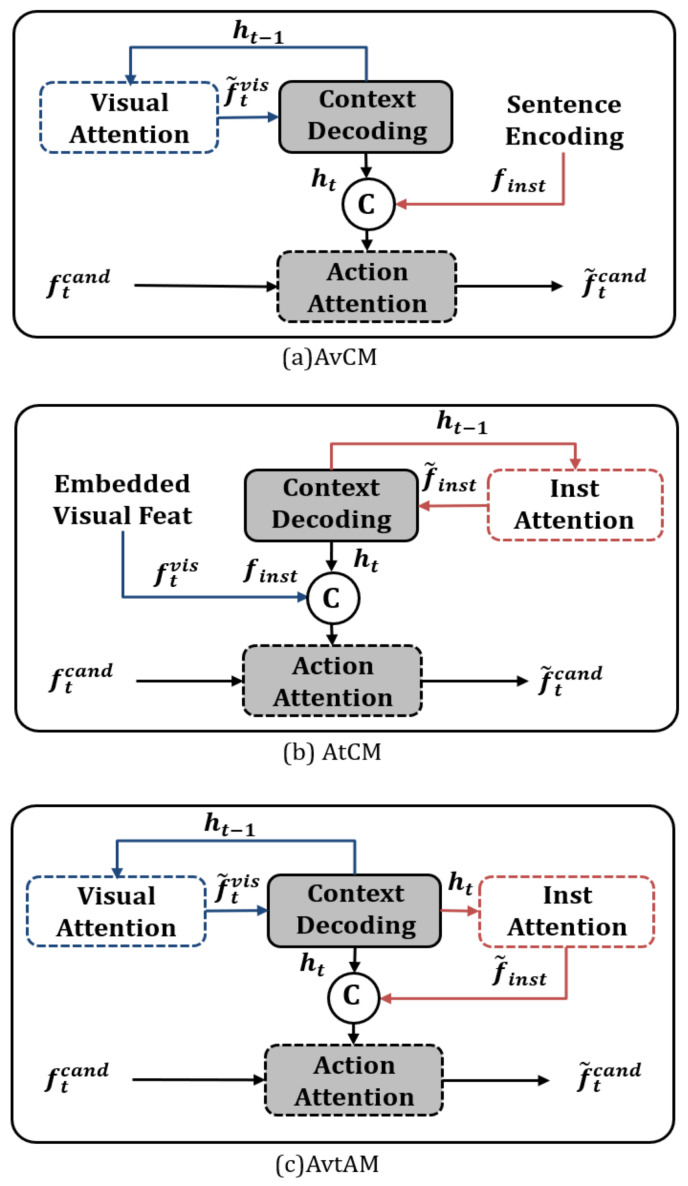
Different temporal contextualization modules: (**a**) attended visual context module (AvCM), (**b**) attended textual context module (AtCM), and the (**c**) attended visual–textual action module (AvtAM).

The second experiment was conducted to prove the positive effect of the attended visual textual action module (AvtAM), the temporal contextualizing module of the proposed model JMEBS. For this experiment, three different implementations of the temporal contextualizing module were compared, as shown in Figure 9. These included the (a) attended visual context module (AvCM (A), (b) attended textual context module (AtCM), and (c) the attended visual–textual action module (AvtAM). Table 2 outlines the experimental results.

The results presented in Table 2 show that AvCM that uses only the visual features (image) to update the temporal context outperformed AtCM that uses only the textual features (instruction) to update the temporal context. Furthermore, AvtAM, the model with the multimodal (visual and textual) attention network used in the model JMEBS, generally scored better than the other models. Conclusively, the temporal contextualizing method (AvtAM) proposed in this study was proven to yield performance improvements.

The third experiment was conducted to prove the superiority of the dynamic time warping (DTW) with triple instructions, the global scoring method used in the proposed model JMEBS. We used the Levenshtein distance (LD) and DTW to measure the similarity of natural language instruction measurements. The partial trajectories executed thus far were checked against (i) single instruction and triple instructions. Table 3 outlines the results of the experiment.

The experimental results in Table 3 show that the triple instructions in both LD and DTW, i.e., LD with triple instructions and DTW with triple instructions, outperformed their single-instruction counterparts in global scoring. Additionally, comparing natural language instructions with word sequences showed that DTW slightly outperformed LD. These research findings verified the superiority of the DTW with triple instructions global scoring method used in the proposed model JMEBS.

The fourth experiment was conducted to prove the search efficiency of the BGLS algorithm proposed in this study. In this experiment, three algorithms were compared: greedy local search, beam search, and BGLS. In the case of the beam search, the number of parallel search trajectories K was varied and took the values of 10, 20, and 30. Table 4 outlines the results of the experiment.

The experimental results in Table 4 show that the search algorithm BGLS proposed in this study has a lower SR than the beam search variations, but outperforms them in all other performance scales. This proves the higher search efficiency of the BGLS compared with the beam search technique. In addition, BGLS showed an improved performance compared with the conventional greedy local search in terms of the SR and NE. These results are interpreted as the positive effect of the backtracking feature that characterized the BGLS.

The last experiment was conducted to prove the superiority of the proposed JMEBS model by comparing it with conventional VLN models. Table 5 outlines the results of the experiment. Among the models compared, the beam search method was applied to tactical-rewind [14], and the greedy local search method was applied to all other models. 

The experimental results in Table 5 show that the proposed JMEBS model outperforms the S-Forcing baseline model [7] by a large margin. For example, JMEBS with backtracking search (BGLS) improves S-Forcing by 35% on success rate (SR), and 33% on success rate weighted by path length (SPL), respectively. Furthermore, our JMEBS model achieves better than or competitive performance against state-of-the-art models. In particular, compared with PREVALENT [20], which is a pretrained multimodal embedding model, JMEBS (Greedy) yields an enhanced performance on SR in both validation-seen and validation-unseen datasets. This result can be interpreted as the positive effect of temporal context used by JMEBS, in addition to the multimodal context. Meanwhile, JMEBS with backtracking search (BGLS) outperforms all models using greedy search, like RCM [18], Self-monitoring [10], Regretful [10], Env-Dropout [15], OAAM [11], and LVERG [12], on success rate. For example, JMEBS (BGLS) improves Regretful [10] by 7% on SR and about 10% on SPL, respectively. On the other hand, although the tactical-rewind model [14] using beam search outperforms other models using greedy local search on SR and NE metrics, it shows worse performance than greedy search-based models and our JMEBS (BGLS) model on SPL because of long search paths. All five types of experiments conducted in this study demonstrated that the proposed JMEBS model leads to significant improvement over existing VLN models.

### 4.3. Qualitative Analysis

**Figure 10:**
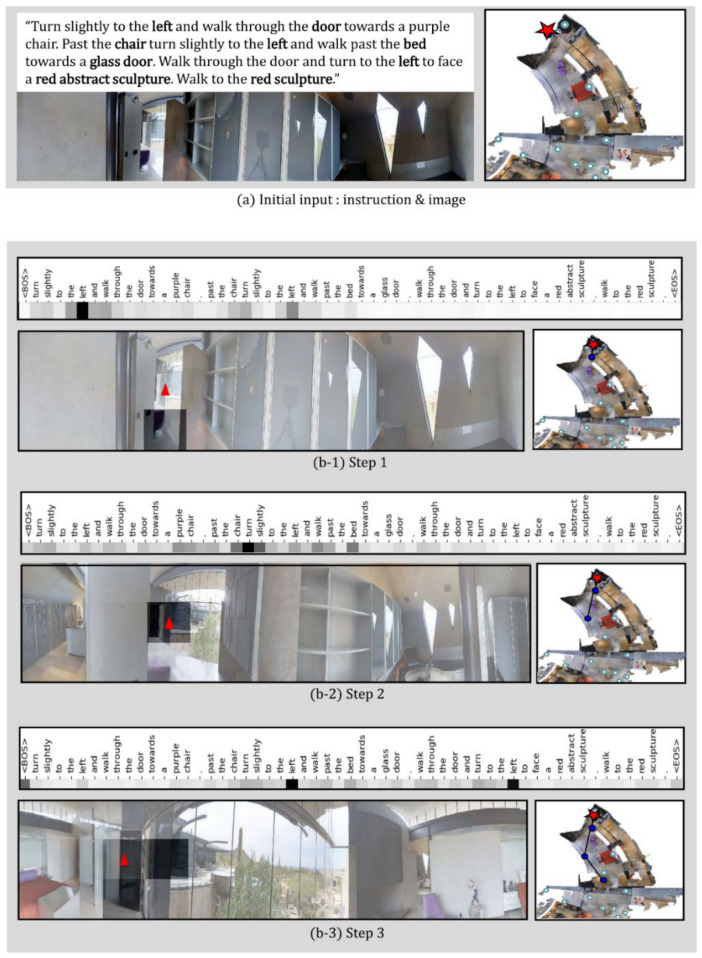
First example of VLN task executed by the proposed model JMEBS: Step1–3.

**Figure 11:**
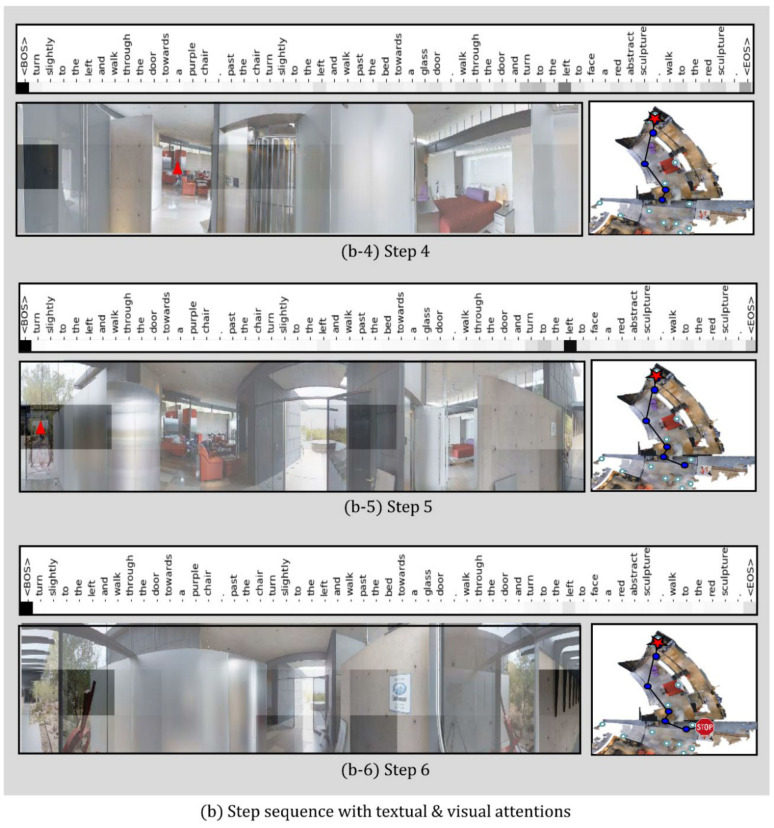
First example of VLN task executed by the proposed model JMEBS: Step 4–6.

**Figure 12:**
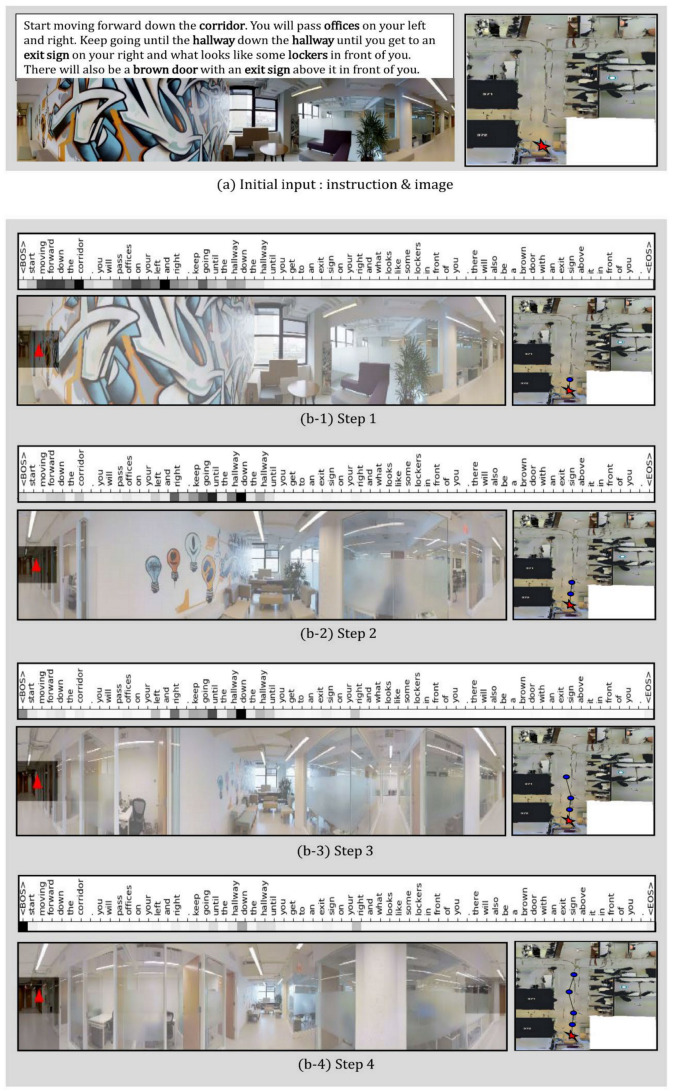
Second example of VLN task executed by the proposed model JMEBS: Step1–4.

**Figure 13:**
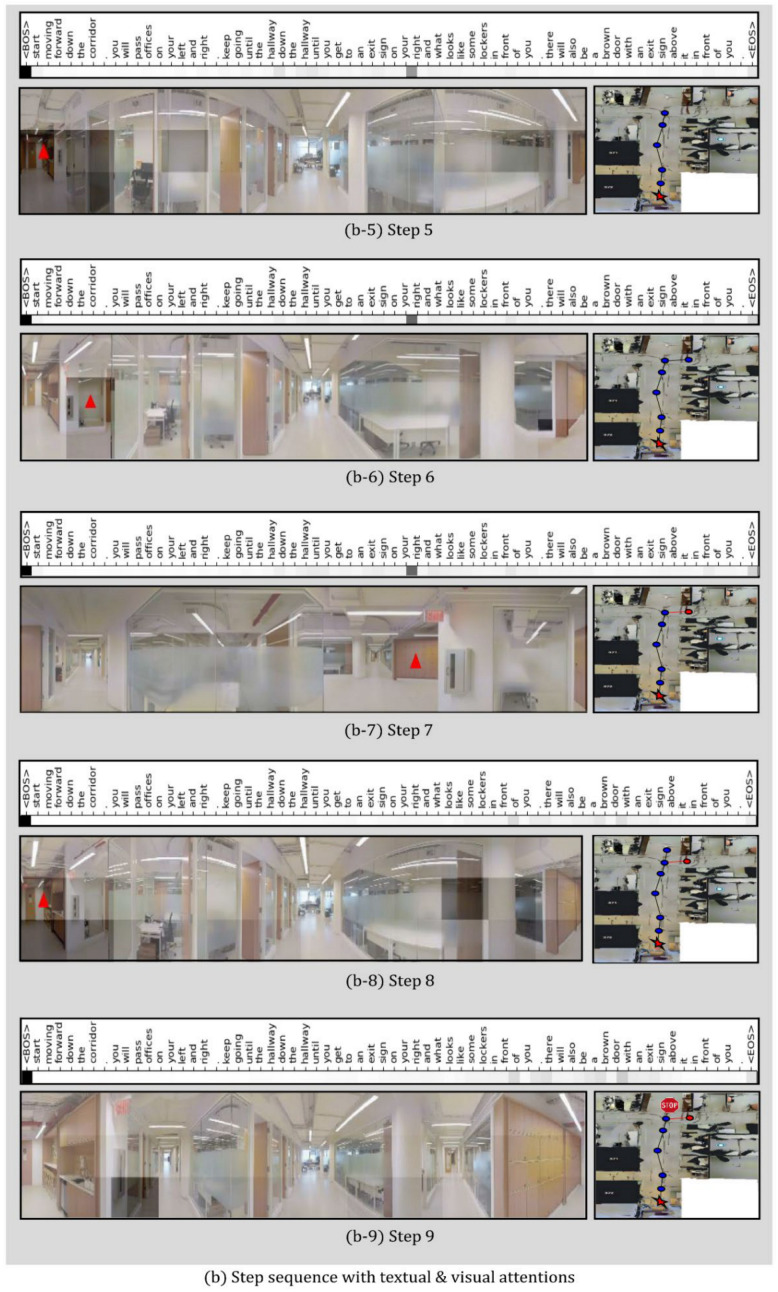
Second example of VLN task executed by the proposed model JMEBS: Step 5–9.

In this study, a qualitative analysis of the proposed JMEBS model was performed on real-life examples of several VLN tasks. Figure 10, Figure 11, Figure 12 and Figure 13 present examples of VLN tasks performed using JMEBS. Each image shows the natural language instruction along with the panoramic image of the agent on the left-hand side and the top-down view of the environment and navigation path on the right-hand side. In particular, the former is marked by current co-attention (textual and visual). The red arrow within the panoramic image represents the agent’s selected navigation action. The textual attention of the natural language instruction and the visual attention of the panoramic image captured by the agent shows that JMEBS has a good perception of the instruction and image areas that require attention at each time step. For example, in steps 2 and 3 in Figure 10, the agent pays attention to the word “left” in the natural language instruction and selects the correct direction by focusing on the left side of the panoramic image. At step 4 in Figure 11, the agent accurately focuses on the area of the “red abstract sculpture” indicated by the natural language instruction and the panoramic image and decides correctly. These results can be interpreted as the high visual–textual alignment and grounding capacity of the JMEBS. Steps 6 and 7 in Figure 13 show an example of the agent’s use of the backtracking feature of BGLS to return to the previous position and take the correct navigation path.

However, JMEBS was observed to pay visual attention to an incorrect image area at step 4 in Figure 11 and step 8 in Figure 13, and textual attention to meaningless tokens, such as the <EOS> and <BOS> mid-path between Figure 10, Figure 11, Figure 12 and Figure 13. These events show that the text–image grounding of JMEBS can be improved further.

## 5. Conclusions

This study proposed the novel deep neural network model JMEBS as an efficient tool to solve VLN tasks. The proposed model was designed to use the past temporal context along with the multimodal context extracted with the joint multimodal embedding module. Additionally, JMEBS employed BGLS, a novel greedy local search algorithm with a backtracking feature that improved the task success rate and search efficiency. The proposed model also came with a new global scoring method for the comparison of all partial trajectories executed to reach the current position with various natural language instructions. Finally, we verified the superiority of the proposed model by various experiments conducted with R2R benchmark datasets.

However, the current JMEBS yielded a slightly worse performance in “unseen” compared to “seen” environments. Previous studies used various methods to improve model performance in unseen environments, such as environmental dropout [15] and self-supervised imitation learning [18]. In this context, improving the generalizability of the JMEBS model to improve its performance in unseen environments would be an important future research project. Furthermore, as verified in the qualitative assessment, there is still scope for improvement for JMEBS in its ability to align and ground natural language instructions and images, despite its use of a pretrained joint multimodal embedding module. We plan to address the drawbacks of the current version of JMEBS in a follow-up study by exploring various methods that add landmark information perceived in the image to the context generation feature in addition to basic visual features.

## Figures and Tables

**Table 1:** Comparison among different embedding features (SR: success rate, SPL: success rate weighted by path lengths, NE: navigation error, JEF: joint embedding features, JEVF: joint embedding visual features, JETF: joint embedding textual features, and JETVF: joint embedding textual and visual features).

Embedding Features	Val-Seen			Val-Unseen		
	SR	SPL	NE	SR	SPL	NE
w/o JEF	62.0	59.0	4.0	52.0	48.0	5.2
JEVF	66.0	61.6	3.6	56.5	50.0	4.5
JETF	66.5	62.9	3.6	55.4	50.9	4.7
JETVF	67.9	63.9	3.6	58.7	53.5	4.4

**Table 2:** Comparison among different temporal contextualization modules.

Temporal Contextualization	Val-Seen			Val-Unseen		
	SR	SPL	NE	SR	SPL	NE
AvCM	67.9	64.1	3.4	58.5	51.8	4.4
AtCM	64.8	60.3	3.6	53.6	49.9	4.8
AvtAM	67.9	63.9	3.6	58.7	53.5	4.4

**Table 3:** Comparison among different global scoring methods.

No.	Global Scoring	Val-Unseen		
		SR	SPL	NE
1	LD with single instruction	54.1	48.9	4.6
2	DTW with single instruction	54.7	49.2	4.6
3	LD with triple instructions	57.1	49.7	4.5
4	DTW with triple instructions	59.0	51.6	4.5

**Table 4:** Comparison among different search methods.

Search	Val-Seen				Val-Unseen			
	SR	SPL	NE	TL	SR	SPL	NE	TL
Greedy	67.9	63.9	3.6	12.1	58.7	53.5	4.4	13.1
Beam (K = 10)	70.3	0.01	5.2	457.9	61.5	0.01	5.2	409.1
Beam (K = 20)	76.6	0.01	4.5	771.2	62.6	0.01	4.6	688.3
Beam (K = 30)	79.2	0.01	4.2	1122.9	72.9	0.01	4.5	1015.2
BGLS	70.1	64.1	3.2	13.5	59.0	51.6	4.4	15.1

**Table 5:** Comparison of the-state-of-art models.

Model	Val-Seen			Val-Unseen			Test		
	SR	SPL	NE	SR	SPL	NE	SR	SPL	NE
Random [7]	15.9	-	9.5	16.0	-	9.2	13.0	12.0	9.8
T-Forcing [7]	27.4	-	8.0	19.6	-	8.6	-	-	-
S-Forcing [7]	38.6	-	6.0	21.8	-	7.8	20.0	18.0	7.8
Speaker-Follower [9]	66.0	-	3.4	36.0	-	6.6	35.0	28.0	6.6
RCM [18]	67.0	-	3.4	43.0	-	5.9	43.0	35.0	6.0
Self-monitoring [10]	67.0	58.0	3.2	45.0	32.0	5.5	48.0	32.0	6.0
Regretful [10]	69.0	63.0	3.2	50.0	41.0	5.3	48.0	40.0	5.7
Env-Dropout [15]	62.0	59.0	4.0	52.0	48.0	5.2	51.5	47.0	5.2
OAAM [11]	65.0	62.0	-	54.0	50.0	-	53.0	50.0	-
LVERG [12]	67.0	70.0	3.47	57.0	53.0	4.7	55.0	52.0	4.7
PREVALENT [20]	67.8	64.8	3.6	57.6	52.2	4.6	54.0	51.0	5.3
Tactical-Rewind [14]	70.0	4.0	3.1	63.0	2.0	4.0	61.0	3.0	4.3
JMEBS (Greedy)	67.9	63.9	3.6	58.7	53.5	4.4	52.1	47.4	5.4
JMEBS (BGLS)	70.1	64.1	3.2	59.0	51.6	4.4	55.7	51.8	5.2

## Data Availability

The datasets used and/or analyzed during the current study are available from the corresponding author on reasonable request.

## Abbreviations

VLN: Vision and language navigation, R2R: room-to-room, VQA: Vision question answering, JMEBS: Joint multimodal embedding and backtracking-enabled search, BGLS: Backtracking-enabled greedy local search, CNN: Convolutional neural network, LSTM: Long short-term memory, BERT: Bidirectional encoder representations from transformers, DTW: Dynamic time warping, LD: Levenshtein distance, AMKM: Attended masked language modeling, AP: Action prediction, NE: Navigation error, SR: Success rate, SPL: Success rate weighted by path length, TL: Trajectory length.
